# Effect of Weather Conditions on the Pediatric Supracondylar Humerus Fracture Incidence

**DOI:** 10.7759/cureus.31558

**Published:** 2022-11-15

**Authors:** Hakan Ozbay, Oktay Adanır, Hamisi M Mraja

**Affiliations:** 1 Orthopaedics and Traumatology, Acıbadem Taksim Hospital, Istanbul, TUR; 2 Orthopaedics and Traumatology, University of Health Sciences, Bağcılar Training and Research Hospital, Istanbul, TUR; 3 Orthopaedics and Traumatology, Istanbul Spine Center & Orthopedics, Group Florence Nightingale Hospitals, Istanbul, TUR

**Keywords:** supracondylar fracture, weather, seasons, incidence, child

## Abstract

Background: Some environmental factors pose as risk factors for children's supracondylar humerus fractures. This study aimed to evaluate the relationship between weather conditions and pediatric supracondylar humerus fracture incidence.

Methods: A total of 310 patients aged <16 years, admitted to our hospital with supracondylar humerus fractures, were evaluated. We evaluated patients' age, and also, season, day, and weather conditions. The Turkish State Meteorological Service database was used for meteorological data and data was analysed statistically.

Results: Most cases occurred in the spring (28.1%, n=87) and summer (27.1%, n=84). Cases of older children (aged six years and older) with supracondylar humerus fractures were recorded in the summer season, while fracture cases in preschool-aged (younger than six years old) children were seen in the winter season.

Conclusion: We found that the overall incidence of pediatric supracondylar humerus fractures increased in spring and summer seasons. In addition, the fracture incidence in preschool- and school-aged children differed according to the season and temperature. Hence, the management of these fractures could also include the significance of weather conditions, making preventive measures more critical in the spring and summer seasons.

## Introduction

Elbow injuries are very common in children and adolescents, and supracondylar fractures of the humerus are the most common elbow fractures in them. They account for 50%-70% of all elbow fractures. Usually, they occur after falling on an outstretched hand [1]. In children, they remain a clinical challenge despite studies on the contributory and protective factors. There are many difficulties in the management of pediatric distal humerus fractures [2]. The effects of weather conditions on the development of these fractures, which are the main cause of childhood orthopedic emergency surgeries, have been controversial. However, the overall fracture incidence among children and the risk of forearm shaft fractures have been higher on dry and warm summer days than on rainy days [3-4].

This study aimed to determine how weather conditions, season, day of the week, and school schedule affected the incidence of pediatric supracondylar humerus fractures.

## Materials and methods

We analysed all the pediatric patients with supracondylar humerus fractures admitted to our emergency department between October 2018 and November 2019. Children younger than 16 years old were evaluated retrospectively. Patients with multiple fractures, open fractures, polytrauma, and ipsilateral injuries were excluded. Our research was conducted in accordance with the principles outlined in the Helsinki Declaration, 2008. Ethical approval was obtained from Bağcılar Training and Research Hospital, Clinical Research Ethics Committee (approval number 2020.10.1.10.156).

Age, sex, fracture type according to the Gartland classification, season, day of the week, and weather condition on admission date were analysed. All patients were divided into two groups: preschool-aged (younger than six years old) and school-aged (six years and older). Also, according to the admission date, two subgroups were developed: in-school days and holidays. Therefore, we aimed to analyse the incidence during in-school days and its relationship with age and weather conditions. The Turkish State Meteorological Service database was used for meteorological data.

Weather analysis was performed by determining the maximum daily temperature on the admission date and subgrouped into cold (<15°C), warm (15-25°C), and hot (>25°C). According to the "millimeter (mm)" value of rainfall, we determined <1 mm of rain as dry and ≥1 mm of rain as wet. The seasons were defined as winter between the winter solstice and the spring equinox, spring between the spring equinox and the summer solstice, summer between the summer solstice and the fall equinox, and autumn between the fall equinox and the winter solstice.

Number Cruncher Statistical Systems (NCSS) 2007 (NCCS, LLC, Kaysville, UT) was used for statistical analysis. Descriptive statistical methods (mean, standard deviation, median, frequency, ratio, minimum, maximum) were used while evaluating the study data. The Pearson chi-square test was used in the comparison of qualitative data. A p-value of <0.05 was considered statistically significant.

## Results

A total of 310 pediatric patients with supracondylar humerus fractures were included; 58.4% (n=181) of patients were male and 41.6% (n=129) were female. The mean age of the patients was 5.35±2.98 years; 62.6% (n=194) of cases were younger than six years old, and 37.4% (n=116) of them were aged six years and older.

Most children with supracondylar humerus fractures were admitted to the emergency room in the spring (28.1%, n=87) and summer (27.1%, n=84) seasons. Other seasons with fracture cases were winter (24.2%, n=75) and autumn (20.6%, n=64). The majority (44.2%, n=137) of fractures occurred on warm days; 28.4% (n=88) of patients were admitted on hot days, while 27.4% (n=85) of cases were admitted on cold days (p<0.01). A total of 83.9% (n=260) of fractures occurred on dry days, while 16.1% of cases occurred on rainy days (n=50). In total, 66.1% (n=205) children with supracondylar humerus fractures were admitted to the emergency room during in-school days, while 33.9% (n=105) cases occurred during the holidays. When we analysed the fracture incidence in children aged six years and older between the holidays and school term period, we found that 70.7% (n=82) cases were admitted to the emergency room on school days. In comparison, 29.3% (n=34) of the fractures occurred during the holidays in this school-aged group.

**Figure 1:**
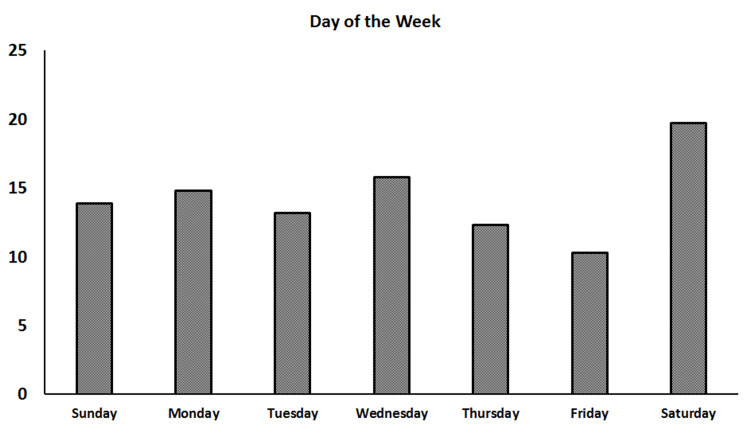
Daily fracture distribution on admission

The days of the week that the majority of fractures were seen were Saturday (19.7%, n=61), Wednesday (15.8%, n=49), and Monday (14.8%, n=46). The daily fracture occurrence distribution during the week is shown in Figure 1.

**Figure 2:**
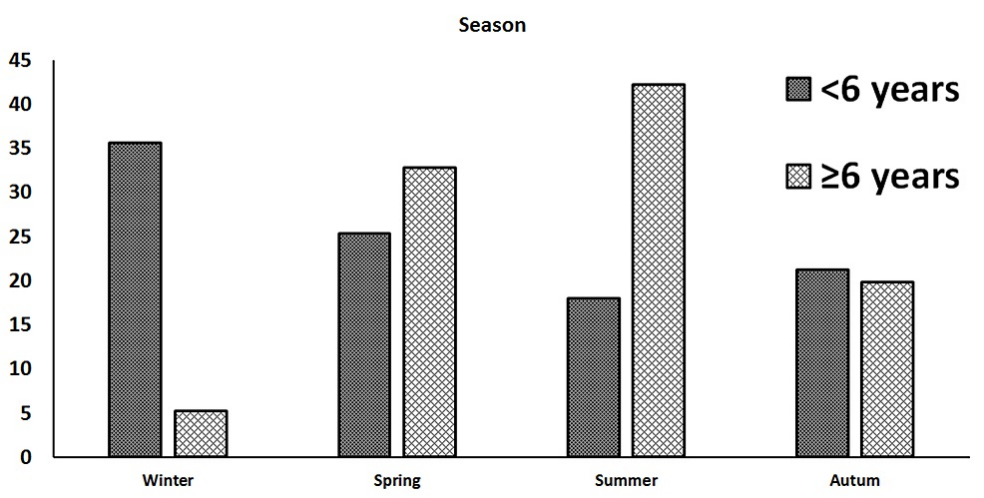
Seasonal distribution of supracondylar humerus fractures between preschool-aged (<6 years) and school-aged (≥6 years) groups

The pediatric supracondylar humerus fracture incidence in school-aged children was higher in the summer season. The majority of preschool-aged children were admitted in the winter season (p<0.01). The seasonal distribution of fracture incidence between preschool- and school-aged groups is shown in Figure 2.

There was a statistically significant difference between seasonal temperature and the age of children. The incidence of supracondylar humerus fractures was higher on warm and hot days in children aged six years and older. Most fractures in preschool-aged children occurred on cold days (p<0.01). There was no statistically significant difference between age groups and rainfall (p>0.05).

## Discussion

Pediatric supracondylar humerus fractures, the most common type of elbow fractures in children, are still challenging in treatment choice or prevention among orthopedic surgeons. Some behavioural and environmental factors have been defined in managing this particular fracture type [1]. One in four children with supracondylar humerus fractures shows some morbidity at long-term follow-ups. Hence, besides the appropriate treatment or other interventions, additional effort could be spent on preventive measures [5].

Pediatric supracondylar humerus fractures are seen most frequently in children between 3 and 10 years of age [1]. Houshian et al. reported that the average age of 355 children with elbow fractures was 7.9 years [6]. Despite most studies, they found elbow fractures more frequent in girls (54%) than in boys. In our study, the mean age of cases was 5.35±2.98 years, and the fracture incidence was higher in boys than girls.

There are studies and controversies about the time of treatment, treatment modality, the configuration of k-wires, and exceptional circumstances like a pulseless hand in pediatric supracondylar fractures. In some areas of North America, this common fracture type is treated by pediatric orthopedic specialists. In 1991, 37% of patients in New England were treated by pediatric orthopedic specialists; by 1999, this figure rose to 68%. Treatment of these fractures is still shifting to the exceptional orthopedic staff worldwide [7]. Also, Scherl et al. stated that preparing trauma units for the summer season is particularly important [8]. In a recent study, Friday was the day of the week when most fractures occurred [9]. Our research found that the incidence of cases increases in warm weather conditions, especially on Saturdays. Also, one in three children with supracondylar humerus fractures was admitted to our hospital on the weekend. Therefore, we think these fractures' complication rates could be diminished by preparing operating rooms and staff, especially on the weekend.

Kao et al. showed the relationship between seasonal temperature and pin site infection incidence [10]. They showed that the rate of pin site infection was significantly higher in the high-temperature season. As shown in our study, the incidence of these fractures increases in high-temperature seasons. We think that orthopedic surgeons can be aware of careful monitoring of infection signs, especially in peak times of these cases. There has been a recent shift toward surgical treatment in pediatric fractures. Some factors, like technological improvements, rapid healing, and minimalization of hospitalization, have strengthened this trend. Environmental and behavioural factors should be understood to prevent and manage these fractures [11].

Play is an essential part of a child's life, helping in the overall development and improving social life of children, but playground equipment should be safe enough to be used by children to prevent injuries. Mott et al. found that approximately 1% of children using playgrounds sustained injuries [12]. The cost of sustaining playground-related injuries remains significant, and preventive measures would be helpful when it comes to financial savings for the individual and the state [13]. Children have an active lifestyle, and when the weather condition is suitable, the incidence of fractures caused by outdoor injuries increases, as shown in our study. During risky weather conditions that might increase these fractures, all supervision and care should be given to the playground environment and equipment. Playground surfaces may be changed to more impact-absorbing material to reduce the incidence and severity of these injuries. Children spend 25%-50% of the daytime in schools depending on age, which is a rationale behind the high risk of a school accident. However, schools are considered safe, and some studies found that the overall annual rate of injury in schools ranges from 2.8% to 9.2% [14]. We found that children aged six years and older were admitted to our clinic on in-school days. We recommend that authorized school staff be aware of this risk, and preventive interventions should be taken to reduce the risk of these fractures.

A recent study that compared public and school playground injury incidence found that the proportion of fractures related to public area playgrounds has decreased significantly with preventive measures. However, it was found that injuries related to school playgrounds increased by 12% [15]. Kraus et al. showed that schoolyard and sports injuries constituted a high percentage of school accidents in a five-year follow-up study [16]. Our study found that the incidence of fractures in children aged six years and older increased during school days. We believe that children in public playgrounds are supervised by parents or babysitters, whereas they are not closely observed in school playgrounds. Mitchelson et al. showed increased complication rates with age [17]. Also, according to our study, if the injuries occurring in a school of children aged older than six years are prevented, incidence and complication rates will decrease. In a study, Song et al. stated that the economic burden increases with age in pediatric fractures [18]. When a high complication rate with increasing age is considered, preventive measures in the school playground gain more importance, especially in these school-aged groups.

There are direct and predictable relationships between risk factors and the incidence of pediatric fractures. The season and climate are risk factors as well. Children living in warmer and colder temperatures have different risk factors in the context of outdoor injuries. Sinikumpu et al. found that dry and warm days are associated with a 2.6- to 3.5-fold higher risk of children's supracondylar humerus fractures compared with rainy or cool days [4]. A study showed a 1.5-fold increase in forearm shaft fracture risk on dry summer days [3]. Unlike these studies from Finland, all four seasons are present distinctly in our geographical area. In a study, Masterson et al. found a strong positive correlation between sunshine hours and fracture admissions [19]. In this study, the average number of fractures in summer was 2.5 times that in winter. In our research, we found pediatric supracondylar humerus fractures occurred on dry and warm days in the spring and summer seasons. Also, we found no relationship between the age of cases and rainy weather conditions. Hot weather is not suitable for outdoor playtime, so orthopedic trauma institutions can be prepared to encounter this fracture type in warm weather conditions. In our study, we found that the incidence of cases was higher in winter at the age of less than six years. Indoor injuries are frequently seen in this age group, which is not a suitable age for outdoor play.

Our study also has some limitations. It is a one-year registry-based study designed in one trauma unit in a local geographical area. Further multi-center studies are needed, including a larger population, with broader periods to understand environmental risk factors in managing and preventing this fracture type. The other limitation is that we did not assess the mechanism of the injury separately.

## Conclusions

Pediatric supracondylar humerus fractures are among the main causes of emergency surgeries in orthopedics. The incidence of supracondylar humerus fractures is higher in dry and warm weather conditions in the spring and summer seasons. Also, the fracture incidence in preschool- and school-aged children differs according to season and temperature. Hence, the management of these fractures could also include the significance of weather conditions, making preventive measures more critical in the spring and summer seasons.
